# A Solvent System Involved Fabricating Electrospun Polyurethane Nanofibers for Biomedical Applications

**DOI:** 10.3390/polym12123038

**Published:** 2020-12-18

**Authors:** Biyun Li, Yinhu Liu, Shuo Wei, Yuting Huang, Shuwen Yang, Ye Xue, Hongyun Xuan, Huihua Yuan

**Affiliations:** School of Life Sciences, Nantong University, Nantong 226019, China; libiyun1986@163.com (B.L.); ericliubio@163.com (Y.L.); weishuo_1205@163.com (S.W.); ann2089166295@163.com (Y.H.); yangshuwen1120@163.com (S.Y.); xuey9@ntu.edu.cn (Y.X.); hyxuan_seu@163.com (H.X.)

**Keywords:** thermoplastic polyurethane, electrospinning, solvent system, controllable mechanical properties, biomedical application

## Abstract

A novel Trichloromethane (TCM)/2,2,2-Trifluoroethanol (TFE) solvent system was developed for fabricating electrospun thermoplastic polyurethane (TPU) nanofibers. TPU solution stability made from this novel solvent system was improved compared to that from the traditional N, N-Dimethylformamide (DMF)/Tetrahydrofuran (THF) solvent system. The minimum TPU solution concentration that can be electrospun was decreased to 0.5% *w*/*v*. The conductivity and viscosity of the TPU solution increased with the increasing ratio of TFE in the solvent system. The obtained electrospun TPU nanofibers fabricated from this novel solvent system showed smooth morphology and uniform diameter distribution. Mechanical strength of TPU nanofibers was improved using this new solvent system. Young’s modulus and tensile strength of the electrospun TPU nanofiber meshes first decreased and then increased, while the strain elongation ratio first increased and then decreased. The new solvent system significantly improves the fiber elongation ratio while maintaining the modulus and tensile strength. The chemical structure of the TPU was not affected by the TCM/TFE solvent system. Electrospun TPU nanofiber meshes prepared by using the TCM/TFE solvent system showed better cytocompatibility, which means the electrospun TPU fibrous scaffold has great potential in biomedical application.

## 1. Introduction

Nanofibers have been widely used in tissue engineering and regenerative medicine [1,2,3], and electrospinning is one of the most convenient and controllable methods to manufacture nanofibers. Thermoplastic polyurethane (TPU) is one type of polymer with excellent properties, such as high strength, high toughness, durable wear resistance and good oil resistance that has been used in textile, food, national defense and other industries [4,5,6]. Because of their excellent biodegradability and biocompatibility, TPU nanofibers have been widely used in biomedical applications [7,8]. Similar to other typical electrospun fibrous scaffolds, many factors have an effect on the application of TPU or TPU-based electrospun fibrous scaffolds, such as the morphology and structure, mechanical properties, and so on. There are many parameters (e.g., rheological properties [9], collection distance and electric field strength [10]) that affect the electrospinning process and the resultant TPU nanofiber morphology. Electrospun solution properties (special for viscoelastic forces) have the most significant influences among these parameters [11].

Electrospun solution properties are directly related to the solvent type. Solvents used to dissolve TPU must be highly polar organic solvents. Currently, several solution systems, mainly composed of 1,1,1,3,3,3,-hexafluoro-2-propanol (HFIP) [12], N,N-dimethyl acetamide (DMAC) [13], dimethyl sulfoxide (DMSO) [13], DMF [14,15,16], DMF/ ethyl acetate [17] or DMF/THF [17,18,19,20], are used for preparing electrospun TPU nanofibers. The effects of several of the above solutions on the resultant TPU nanofibers have been investigated by Mondal [13] and Cay et al. [17]. These studies demonstrated that the morphology and properties of the resultant TPU nanofibers have been changed significantly with the solvent selection. DMF or DMF-based solvent seemed to be the best solvent among the above-used solvents to prepare electrospun TPU solution. However, DMF solvent is difficult to volatilize and is toxic. DMF remains in the prepared electrospun TPU fiber, which can induce the cytotoxicity of TPU nanofiber scaffold. Therefore, it is necessary to develop a novel, highly polar and volatile solution system for the preparation of TPU nanofibers to reduce solvent residue. TCM and TFE are two types of highly polar and volatile organic solvents. However, there are no reports on the preparation of TPU nanofibrous scaffolds using these two solvents.

Herein, we fabricated TPU nanofibers from different solvent combinations (TCM and TFE). The manufactured nanofibrous meshes were then characterized by field emission scanning electron microscopy (FE-SEM), Fourier transform infrared spectroscopy (FT-IR), X-ray diffraction (XRD), differential scanning calorimetry (DSC) and mechanical property tests. The in vitro cytotoxicity of prepared nanofibers was tested using rat bone marrow mesenchymal stem cells (rMSCs).

## 2. Materials and Methods

### 2.1. Materials

Thermoplastic polyurethane (TPU, 1185A, 1.12 g/cm^3^) was obtained from BASF (Germany). 2,2,2-Trifluoroethanol (TFE, purity ≥ 98%, Shanghai Darui Fine Chemicals, Shanghai, China), dimethylformamide (DMF, purity ≥ 98%, Shanghai Aladdin Bio-Chem Technology Co., Ltd., Shanghai, China), tetrahydrofuran (THF, Shanghai Ling Feng Chemical Reagent Co., Ltd., Shanghai, China) and trichlormethane(TCM, Yonghua Chemical Co., Ltd., Changshu, Jiangsu, China) were used as solvents to dissolve TPU. These materials and chemicals were used as received without further purification.

### 2.2. DMF/THF and TCF/TFE Solution Systems

TCM and TFE were mixed at the volume ratio of 10:0, 9:1, 8:2, 7:3, 5:5, 4:6, 3:7 and 0:10. Then, the same amount of TPU was added to every mixture to prepare a spinning solution at a concentration of 0.5%, 5% and 8% (*w*/*v*), respectively. The mixture was stirred using a magnetic bar until the solution was uniform and free of bubbles. Based on previous studies [21,22] DMF and THF mixed at the volume radio of 5:5 was used as the control group.

The viscosity of the above solution was tested using the ndj-8s digital viscometer (Shanghai lichen bangxi instrument technology Co., Ltd., Shanghai, China). The measurement temperature was between 24 and 26 °C, and rotor speed was 60 rpm. The conductivity of the solutions was measured by DDS-307A conductivity meter made by Shanghai Instrument & Electronics Scientific Instrument Co., Ltd., Shanghai, China.

### 2.3. TPU Nanofibers Preparation

Applied voltage of the electrospinning setup was 10–16 kV. The tip-to-collector distance was 10–15 cm. The feeding rate was set at 1 mL/h. The relative humidity of the environment was 30–60%. All the experiments were conducted at room temperature. The TPU nanofiber meshes obtained from electrospinning were dried in a vacuum-drying oven for 48 h to remove the residual organic solvent.

### 2.4. Characterization

A field emission scanning electron microscope (FE-SEM, ZEISS Gemini SEM 300, Oberkochen, Germany) was used to characterize electrospun nanofiber morphology. The accelerating voltage was set at 8–10 kV. The diameter of electrospun nanofibers was measured by Image J 1.40G software, and the average diameter of nanofibers in each solution system was calculated. The number of nanofibers in each calculated group from each solution system was above 50.

A TENSOR 27 FT-IR spectrometer (Bruker, Karlsruhe, Germany) was used to characterize the molecular information of the electrospun nanofibers. The FT-IR spectra of the electrospun nanofibers were obtained over the range of 600–4000 cm^−1^ at a scanning resolution of 2 cm^−1^.

To examine the effect of a different volume ratio of TCM and TFE on the crystalline characteristics of electrospun nanofibers, XRD analysis was performed using a Rigaku D/max 2550 PC with Cu Ka radiation, operated at 40 kV, 300 mA, and a 5° (2θ) per min scanning rate.

DSC (204 F1, Netzsch, Selb, Germany) analysis was also performed to examine the crystalline information of the electrospun nanofibers. Each sample weighed between 5 to 8 mg. The instrument was calibrated with an indium standard. A nitrogen atmosphere (flow rate is 50 mL/min) was used throughout the scanning. The experiment was conducted from −20 to 270 °C at a heating rate of 10 °C/min.

Tensile properties of the electrospun nanofibrous meshes were tested using a tabletop testing machine (ZQ-990LB, Zhichao Precision Instrument Co., Ltd., Nanjing, China) equipped with a 500 N load cell. Rectangular-shaped specimens with a size of 50 mm (length) × 10 mm (wide) × 0.1−0.2 mm (thickness) were stretched at a constant cross-head speed of 10 mm/min at room temperature. Typical tensile properties of strength, Young’s modulus and strain-at-break were obtained from the tensile stress–strain curves.

### 2.5. Cytotoxicity Assay

The TPU solution was dissolved in the TCM/TFE and DMF/THF system at a concentration of 8% (*w*/*v*). The solutions were stirred overnight till uniform and bubble-free. The electrospun nanofiber meshes were dried in a vacuum oven to remove the solvent completely. The nanofiber meshes were used to cover a coverslip with a 15 mm diameter. After that, the nanofiber meshes and coverslip were soaked in alcohol solution (100%, 90%, 80%, 70%, 60%, 50%) from high-concentration to low-concentration for 15 min each and then washed twice in PBS (phosphate buffer saline) solution to complete the disinfection and sterilization. The 24 well plates were used for the cell test. There were five control groups with three multiple holes in each group. rMSCs were cultured in DMEM (Hyclone, South Logan, UT, USA) supplemented with 10% FBS (Zhejiang Tianhang Biotechnology Co. Ltd., Huzhou, China) and 1% penicillin/streptomycin (Tianjin Haoyang Biological Products Technology Co. Ltd., Tianjin, China) for 7 days (37 °C, 5% CO_2_). The medium was changed every 2 days. Then, the cells were isolated by 0.125% trypsin-EDTA solution (Tianjin Haoyang Biological Products Technology Co. Ltd., China) and counted using a hemocytometer and an optical microscope.

After 4 and 7 days of cell culture, specimens were fixed by 0.25% glutaraldehyde solution for 4 h, followed by dehydration through a graded series of alcohol solution concentrations (50%, 60%, 70%, 80%, 90%, 100%) for 15 min each time. Thereafter, the specimens were dried and mounted on aluminum holders and coated with gold for FE-SEM imaging.

Cells were seeded onto each TPU fibrous membrane at a density of 1 × 10^4^ cells per well and cultured in an incubator. Cell viability was tested using CCK-8 assays (Beyotime Biotechnology Co. Ltd., Shanghai, China).

### 2.6. Statistical Analysis

All values were exhibited as the mean ± standard error of at least three samples. Statistical analysis was performed using one-way ANOVA analysis with Tukey’s test included in Origin 8.0 software. *p* < 0.05 was considered statistically significant.

## 3. Results and Discussion

### 3.1. Solution Properties of TPU in TCM/TFE Solvent Systems

The TPU solution properties are the most important parameters that affect the resultant TPU nanofibers properties [13]. Previous studies have demonstrated that TPU can be easily dissolved in polar solvents [13,17], so we chose two polar solvents, TCM and TFE. The polarity of the mixed solvent depends on the ratio of the two solvents. The solubility of TPU in the TCM/TFE solvent system with different solvent ratios is shown in Table 1. When the concentration of TPU was 5% (*w*/*v*), TPU only swelled at V_TCM_/V_TFE_ = 10/0, 9/1, 3/7 and 0/10. TPU can be dissolved and form a uniform and stable TPU solution when V_TCM_/ V_TFE_ is 8/2, 7/3, 6/4, 5/5, 4/6. These solutions keep clear and transparent during long-term storage, and this is beneficial to industrial production.

**Figure 1:**
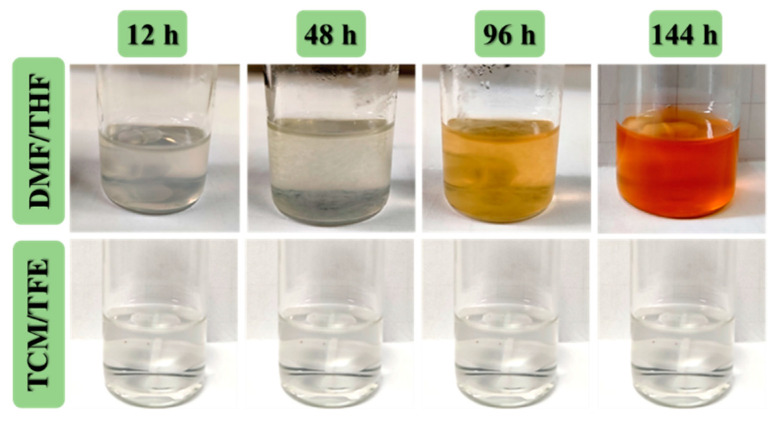
Stability of TPU (5% *w*/*v*) solutions at different solvents.

DMF and THF were common solvents of TPU in previous studies [21,22,23]. In this study, the DMF/THF (5/5) solvent system was used as the control group. Compared with the TPU solution made from DMF/THF, TPU solution made from the TCM/TFE (5/5) solvent system was much more stable. As shown in Figure 1, TPU flocculent precipitate appeared after 48 h of standing in the DMF/THF solvent system, and the solution gradually turned orange-red. It can be concluded that TPU is unstable in the DMF/THF solvent system. However, TPU can quickly dissolve in the TCM/TFE solvent system. Moreover, the TPU (TCM/TFE) solution system is homogeneous and stable. No precipitates or color change happened in this solvent system, which suggests that the chemical structure of TPU is stable in this novel solvent system.

### 3.2. Electrospun TPU Nanofibers

**Figure 2:**
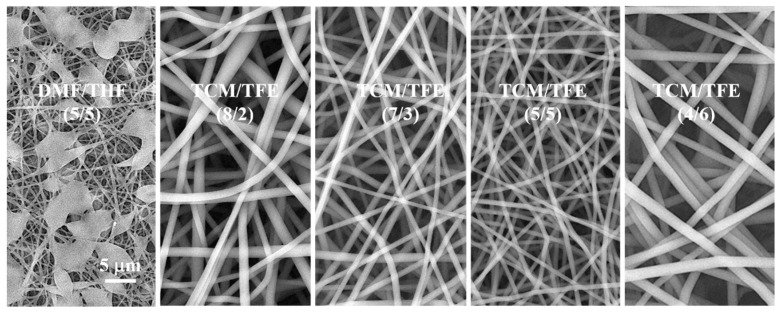
SEM images of electrospun TPU nanofibers from DMF/THF and TCM/TFE solvent systems at different ratios of solvents (5% *w*/*v*).

It is well-known that polymer concentration plays an important role in maintaining the stability of electrospun jet [9]. When the concentration of the solution is lower than the optimum concentration, polymer chains in the solution do not have enough crosslinks. This will make it difficult to form a Taylor cone or get electrospun fibers with beads while performing the electrospinning. Figure 2 shows SEM images of nanofibers prepared by electrospinning from TCM/TFE and DMF/THF solvent systems with TPU concentration of 5% (*w*/*v*). Electrospun TPU nanofibers from TCM/TFE solvent show a smooth surface morphology and uniform diameter distribution, while electrospun TPU nanofibers from DMF/THF show many beads. There are several reasons for the formation of beads. One is the low solubility of TPU in the DMF/THF solvent, which results in the low crosslinks among the TPU polymer chains in the solution. The other is from the low volatility of DMF (saturated vapor pressure of 3.7 mmHg) in the DMF/THF solvent system. In this case, the solvent cannot completely volatilize, while the drops fly from the needle tip to the collector. Compared to DMF, TCM has a much higher volatility (saturated vapor pressure is 160 mmHg) [17]. Even if the concentration of TPU in the solvents of TCM/TFE is decreased to a minimum electrospinnable concentration of 0.5% (*w*/*v*), the solvent can volatilize in time and facilitate nanofibers formation. Therefore, TCM/TFE solvents improved the electrospinnability of TPU, which is beneficial for reducing the production cost and its use as a biological scaffold.

**Figure 3:**
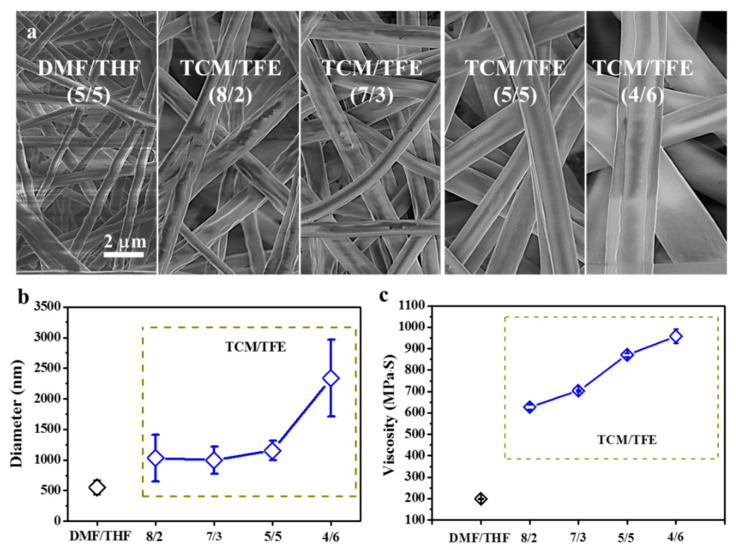
(**a**) SEM images and (**b**) diameters of electrospun TPU nanofibers from DMF/THF and TCM/TFE solvent systems; (**c**) viscosities of TPU in DMF/THF and TCM/TFE solvent systems (8% *w*/*v*).

When the TPU concentration was increased to 8% (*w*/*v*), TPU solutions from both TCM/TFE and DMF/THF solvent systems could be electrospun into nanofibers with a smooth surface and uniform diameter distribution (Figure 3). The TPU fiber diameter from DMF/THF solvent is (549 ± 117) nm and the TPU fiber diameter from TCM/TFE solvent is (1155 ± 161) nm. The diameter of the nanofibers produced by the DMF/THF solvent system is significantly smaller than that of TCM/TFE. This is mainly because the viscosity of TPU-DMF/THF solution is much lower than that of TPU-TCM/TFE. Figure 3a shows SEM images of electrospun TPU nanofibers from TCM/TFE and DMF/THF solvent systems (TPU concentration is 8% *w*/*v*). TPU nanofibers prepared by electrospinning with different solvent ratios of TCM/TFE have smooth and bead-free morphology. As the TFE component in the solvent system increases, the surface roughness of the fiber first decreases and then increases. TPU nanofibers have the lowest surface roughness when the ratio of TCM/TFE is V_TCM_/V_TFE_ = 5/5. Diameters of the nanofibers were (1032 ± 380) nm, (997 ± 226) nm, (1155 ± 161) nm, (2339 ± 628) nm for V_TCM_/V_TFE_ = 8/2, 7/3, 5/5, 4/6, respectively (Figure 3b). The diameter of TPU nanofibers increased the most when the TCM/TFE ratio changed from 5/5 to 4/6. The viscosity of TPU solution in DMF/THF and TCM/TFE solvent systems are shown in Figure 3c. It can be concluded that the viscosity of TPU solution in TCM/TFE solvent system increased with the increase of TFE ratio. During the formation of electrospun nanofibers, the most critical factor affecting the fibers’ diameter is the splitting of droplets after leaving the nozzle in the electric field [3]. If the droplet can be easily split, the nanofibers with fine fiber diameters can be obtained. On the contrary, the more difficult it is for droplets to be split, the larger the diameter of the nanofibers obtained. Previous research [24] reported that the solution viscosity, volatility of solvent system and conductivity have a great influence on the diameter and morphology of nanofibers. It can be inferred that when V_TCM_/V_TFE_ = 4/6, the extremely significant increase in the diameter of the nanofiber is caused by the increase in the viscosity of the solution, which means the viscosity of the solution becomes the main factor affecting the fiber diameter.

**Figure 4:**
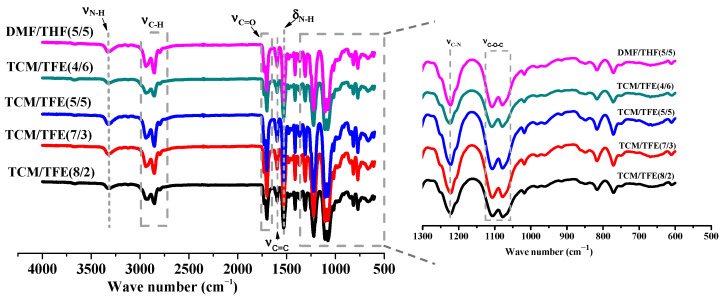
FT-IR spectra of TPU nanofibers from TCM/TFE and DMF/THF solvent systems.

The molecular structure of electrospun TPU nanofibers from DMF/THF and TCM/TFE solvent system was characterized by Fourier transform infrared spectrometer (FTIR), as shown in Figure 4. The repeated amide group is a characteristic group of TPU [25]. It can be seen from the infrared absorption spectra that there is no significant difference in the chemical groups between TPU nanofibers from DMF/THF and TCM/TFE solvent systems. In general, the typical absorption peaks of the amide bond in the TPU chemical structure can be detected at 1700 cm^−1^ (stretching vibration of C=O in amide bond), 1530 cm^−1^ (bending vibration of amide bond N-H) and 1223 cm^−1^ (C–N stretching vibration of amide bond) [20]. It can be concluded that the solvent in the solution system will not change the chemical structure of TPU. In addition, other chemical groups of TPU can also be observed in the infrared absorption spectrum, such as the asymmetric stretching vibration of CH_2_ at 2929 cm^−1^ [15], the stretching vibration of symmetrically distributed CH_2_ at 2830 cm^−1^, and the C=C skeleton vibration of aromatic benzene ring at 1600 cm^−1^. In the infrared absorption spectrum, the stretching vibration of C=O bond from the ester group at 1730 cm^−1^ and the stretching vibration of C–O–C at 1100 cm^−1^ were also observed [26]. However, due to vibration coupling, there was a peak before and after 1100 cm^−1^.

**Figure 5:**
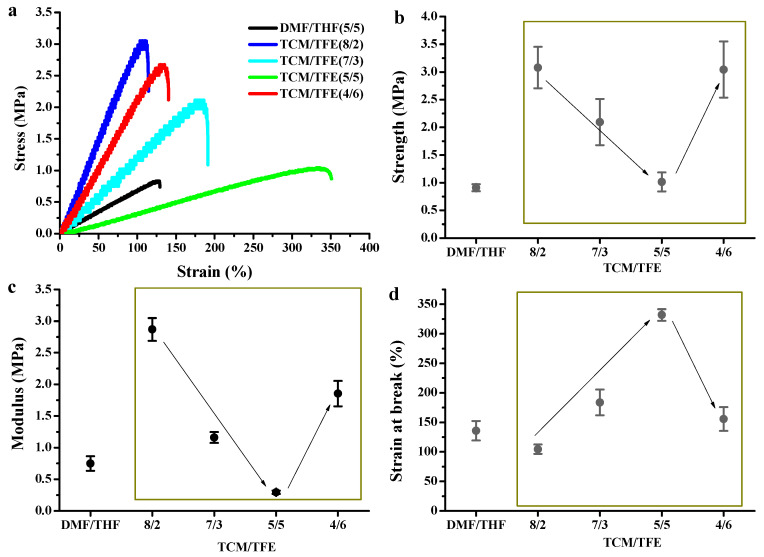
Mechanical properties of TCM/TFE and DMF/THF electrospun nanofibers: (**a**) typical stress-strain curves, (**b**) tensile strength, (**c**) modulus, (**d**) strain at break.

Figure 5 shows the mechanical properties of electrospun TPU nanofibers manufactured from TCM/TFE and DMF/THF systems. With the increase of TFE in the solvent system, the tensile strength of TPU nanofibers (Figure 5b) first decreased and then increased. The tensile strength of TPU nanofibers reaches the lowest value when V_TCM_/V_TFE_ is 5/5. The tensile strengths were 3.08, 2.09, 1.01, 3.04 MPa for V_TCM_/V_TFE_ = 8/2, 7/3, 5/5 and 4/6, respectively. With the increase of TFE in the solvent system, the Young’s modulus of electrospun TPU nanofibers (Figure 5c) first decreased and then increased. The Young’s modulus reached the lowest value at V_TCM_/V_TFE_ = 5/5. Young’s moduli were 2.87, 1.16, 0.30 and 1.86 MPa for V_TCM_/V_TFE_ = 8/2, 7/3, 5/5 and 4/6, respectively. With the increase of TFE in the system, the strain elongation ratio of TPU fibers from TCM/TFE solvent system first increased and then decreased. The strain elongation ratio reached the highest value when the ratio of V_TCM_/V_TFE_ was 5/5. The strain elongation ratio of the TPU fibers from each solvent system showed an opposite trend to the tensile strength of the fiber mats. The strain elongation ratios were 104.51%, 183.65%, 331.79% and 155.70%for V_TCM_/V_TFE_ = 8/2, 7/3, 5/5 and 4/6, respectively. Mechanical properties of TPU nanofibers are highly related to the TCM and TFE combinations. TCM is highly volatile, but TFE is less volatile than TCM. With the increase of the TFE ratio in the solvent system, the volatility of TCM/TFE solution prepared for electrospinning became weaker. Therefore, there is still a little solvent left when the fiber reached the collecting plate, and this promoted more crosslinking of the fiber, thus enhancing the maximum elongation of the fiber.

**Figure 6:**
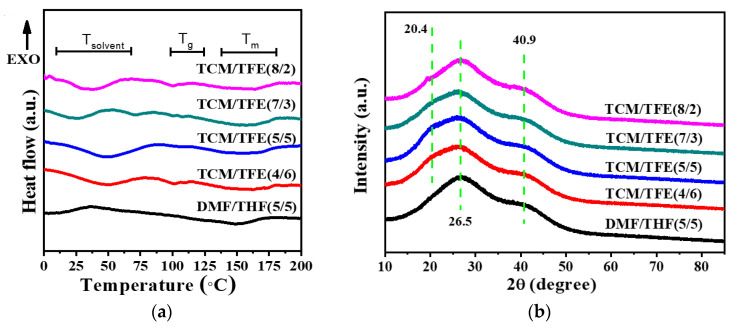
(**a**) Thermal properties of electrospun TPU nanofibers by DSC; (**b**) XRD patterns of TPU nanofibers.

Figure 6a shows the thermal properties of TPU nanofibers. The specific solvent evaporation temperature, melting temperature and glass transition temperature are listed in Table 2. All samples show a broad endothermal peak from 10 to 70 °C. This peak is attributed to both the evaporation of the solvent (TCM, TFE and THF) residue and the melting of the soft segment of TPU [27]. The broad endothermal peak around 162 °C was from the melting of the hard segment in TPU nanofiber meshes from the TCM/TFE solvent system, while the counterpart of the TPU nanofibers mesh from the DCM/THF solvent was around 150 °C. This suggests that TPU nanofiber meshes from the new solvent system show better thermal stability. TPU nanofiber meshes from the TCM/TFE solvent system showed a glass transition of the hard segment of TPU around 102 °C, while the *T*_g_ of the TPU nanofiber from DMF/THF solvent showed a less weak glass transition around 103 °C. This suggests that the TPU nanofiber from TCM/TFE solvent system had more hard segments [28], and thus the TPU nanofiber mesh from TCM/TFE solvent system had a higher Young’s modulus. However, the sample from the TCM/TFE (5/5) solvent system showed the weakest glass transition around 102 °C, which suggests that it had the lowest hard segments among all the samples from the new solvent system. This also accounts for the lowest Young’s modulus of the TCM/TFE (5/5) sample. This finding should inspire researchers, as it means that the ratio of soft segments and hard segments is tunable through modifying the new solvent system.

Figure 6b shows the XRD spectra of electrospun TPU nanofibers. TPU nanofibers from TCM/TFE solvent systems all display a strong diffraction peak at 25.9° with two shoulders at 20.4° and 40.9°. The peak at 20.4° represents the existence of a mixed ordered structure of both hard and soft domains and an amorphous phase of the TPU matrix [29]. The peaks at 25.9° and 40.9° represent the mixing part of hard and soft components of TPU, respectively [30]. There is no shoulder at 20.4° for the TPU nanofibers from DMF/THF solvent system. This implies that the crystalline properties of TPU may be modified by different solvent systems, and this is consistent with the results of DSC.

**Figure 7:**
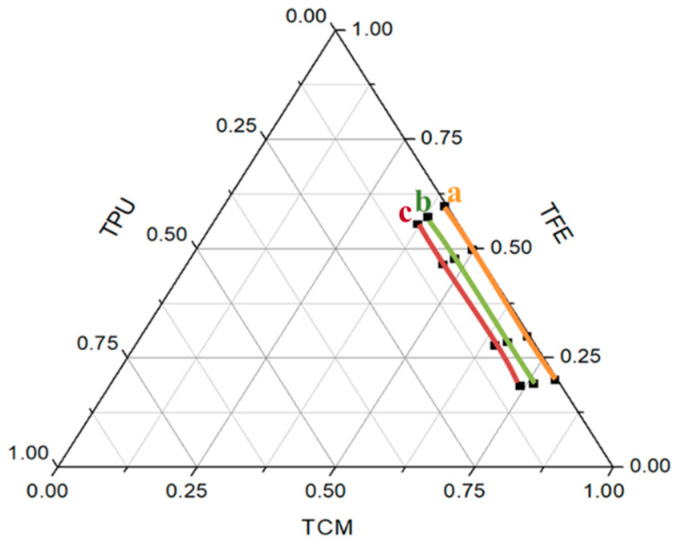
Mapping of the region for the production of bead free TPU fibres with: (**a**) 0.05% *w*/*v*, (**b**) 5% *w*/*v*, (**c**) 8% *w*/*v*. The area enclosed by the three colored lines indicates the ternary mixture compositions that can lead to the production of bead-free TPU nanofibers.

The ternary phase diagram can clearly show the ratio range of the solvent, which can produce uniform and smooth fibers without liquid beads in theTCM/TFE solvent system [31]. As shown in Figure 7, smooth, uniform and non-adhesive nanofibers can be obtained with TPU concentrations ranging from 0.5% to 8% and TCM accounting for 80% to 40% of the solvent system. According to previous research, when DMF and THF mixed solution is used as solvent, the required concentration of polyurethane for electrospinning is 6%–18% [22,23]. As shown in Figure 7, compared with the DMF/THF solvent system, smooth and uniform nanofibers can be obtained by electrospinning with smaller TPU polymer concentration and more solvent ratio by using the TCM/TFE solvent system.

**Figure 8:**
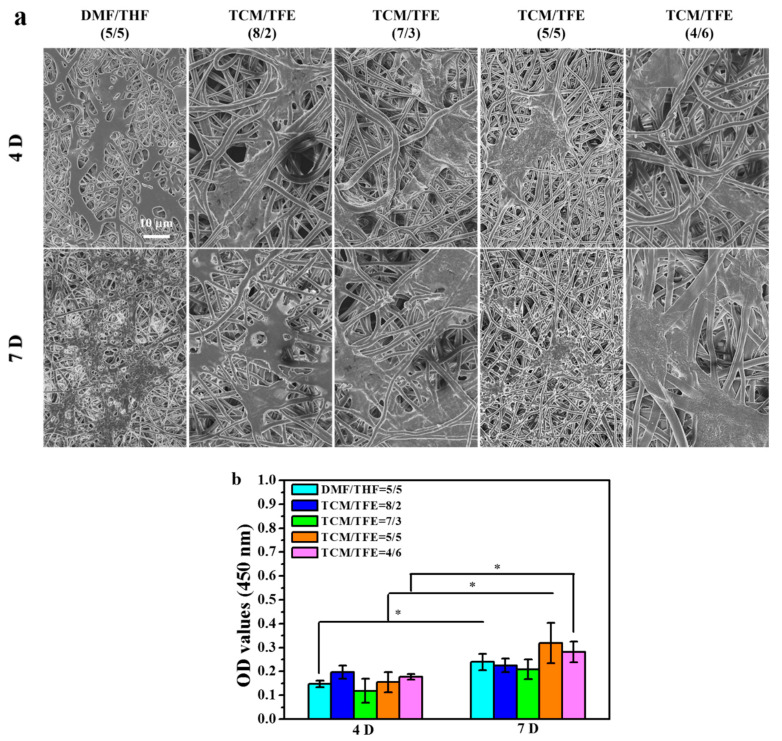
(**a**) SEM images of rMSCs cultured on electrospun TPU nanofiber meshes; (**b**) cell viability of rMSCs on TPU nanofiber scaffold at day 4 and day 7. * suggests these data were considered statistically significant (*p* < 0.05).

Figure 8a shows SEM images of the rMSCs on the electrospun TPU nanofiber scaffolds. At day 4 of culture, the TPU nanofiber scaffolds from both DMF/THF and TCM/TFE solvent systems show close cell viability. It can be clearly seen that the TPU nanofiber scaffolds provide solid support for the rMSCs and that the cell adhered well to the scaffold. At day 7 of culture, rMSCs distributed uniformly on the TPU nanofiber scaffold from TCM/TFE at V_TCM_/V_TFE_ = 5/5 solvent while gathering close to each other on other samples. The quantitative cell viability of rMSCs was measured by CCK-8 method. In particular, at day 7, there was nearly 1.3-fold cell growth on the TPU nanofiber scaffold from TCM/TFE at V_TCM_/V_TFE_ = 5/5 than that from DCM/THF at V_DCM_/V_THF_ = 5/5. We can also easily see that the TPU nanofiber scaffold from TCM/TFE at V_TCM_/V_TFE_ = 5/5 solvent showed the highest OD values (Figure 8b). This is because the TPU nanofibers from V_TCM_/V_TFE_ = 5/5 solvent have a smaller diameter. In this case, the scaffold has a higher porosity, and this is beneficial to providing sufficient culture nutrition for the rMSCs. These data suggest that the TPU scaffolds from the solvent system of TCM/TFE (5/5) show excellent biocompatibility.

## 4. Conclusions

A novel solvent system with TCM and TFE at different component ratios were developed for fabricating electrospun TPU nanofibers. The selected TCM and TFE component ratios are V_TCM_: V_TFE_ = 8:2, 7:3, 5:5, 4:6. The concentration of TPU ranges from 0.5% to 8%. The obtained electrospun TPU nanofibers fabricated from this novel solvent system showed smooth morphology and uniform diameter distribution. The viscosity of the TPU solution increased with the increasing ratio of TFE in the solvent system. The Young’s modulus and tensile strength of the electrospun TPU nanofiber meshes first decreased and then increased. The maximum tensile strength of the electrospun TPU nanofiber first increased and then decreased. FTIR data showed that the chemical structure of the TPU was not affected by the TCM/TFE solvent system when compared to that of the DMF/THF solvent system. The TPU solution stability from the TCM/TFE solvent system was improved in contrast with the DMF/THF solvent system. The new solvent system can significantly improve the fiber tensile elongation ratio while maintaining the modulus and tensile strength. Electrospun TPU nanofiber meshes prepared using the novel TCM/TFE solvent system did not show cytotoxicity, which means the electrospun TPU meshes have excellent biocompatibility.

## Figures and Tables

**Table 1:** Solubility of TPU (5% *w*/*v*) in TCM/TFE solvent system at different solvent ratios.

V_TCM_: V_TFE_	10:0	9:1	8:2	7:3	6:4	5:5	4:6	3:7	0:10
**Swelling**	**✓**	**✓**	**✓**	**✓**	**✓**	**✓**	**✓**	**✓**	**✓**
**Dissolved**	**✕**	**✕**	**✓**	**✓**	**✓**	**✓**	**✓**	**✕**	**✕**

**Table 2:** Thermal properties of TPU nanofibers.

	8/2	7/3	5/5	4/6	DMF/THF
**T_solvent_**	37.2 °C	23.8 °C	52.1 °C	49.6 °C	15.2 °C
**T_g_**	101.6	102.2	102.7	103.2	103.7
**T_m_**	162.6 °C	161.1 °C	160.2 °C	163.1 °C	150.7 °C
